# Automated image acquisition and analysis of graphene and hexagonal boron nitride from pristine to highly defective and amorphous structures

**DOI:** 10.1038/s41598-024-77740-9

**Published:** 2024-11-06

**Authors:** Diana Propst, Wael Joudi, Manuel Längle, Jacob Madsen, Clara Kofler, Barbara M. Mayer, David Lamprecht, Clemens Mangler, Lado Filipovic, Toma Susi, Jani Kotakoski

**Affiliations:** 1 Faculty of Physics, University of Vienna, Boltzmanngasse 5, 1090 Vienna, Austria; 2 Vienna Doctoral School in Physics, University of Vienna, Boltzmanngasse 5, 1090 Vienna, Austria; 3 Institute for Microelectronics, TU Wien, Gusshausstrasse 27-29/E360, 1040 Vienna, Austria

**Keywords:** Graphene, Imaging techniques

## Abstract

Defect-engineered and even amorphous two-dimensional (2D) materials have recently gained interest due to properties that differ from their pristine counterparts. Since these properties are highly sensitive to the exact atomic structure, it is crucial to be able to characterize them at atomic resolution over large areas. This is only possible when the imaging process is automated to reduce the time spent on manual imaging, which at the same time reduces the observer bias in selecting the imaged areas. Since the necessary datasets include at least hundreds if not thousands of images, the analysis process similarly needs to be automated. Here, we introduce disorder into graphene and monolayer hexagonal boron nitride (hBN) using low-energy argon ion irradiation, and characterize the resulting disordered structures using automated scanning transmission electron microscopy annular dark field imaging combined with convolutional neural network-based analysis techniques. We show that disorder manifests in these materials in a markedly different way, where graphene accommodates vacancy-type defects by transforming hexagonal carbon rings into other polygonal shapes, whereas in hBN the disorder is observed simply as vacant lattice sites with very little rearrangement of the remaining atoms. Correspondingly, in the case of graphene, the highest introduced disorder leads to an amorphous membrane, whereas in hBN, the highly defective lattice contains a large number of vacancies and small pores with no indication of amorphisation. Overall, our study demonstrates that combining automated imaging and image analysis is a powerful way to characterize the structure of disordered and amorphous 2D materials, while also illustrating some of the remaining shortcomings with this methodology.

## Introduction

In recent years, the field of two-dimensional (2D) materials research has moved in a direction where not only pristine lattices, but defects and imperfections are intensively studied. Vacancies, topological defects or grain boundaries impact the material properties, sometimes even dominating them. For example, it was shown that point defects in hexagonal boron nitride (hBN) can be used as quantum emitters^1–7^, which are particularly interesting due to their stability over a wide temperature range^8^, as well as their bright emission into the zero-phonon line^9^, the wide spectral range they cover^2,4,10^, and the possibility for lifetime-limited emission at room temperature^11,12^. Following experiments in which graphene was amorphized using electron irradiation^13,14^, even the synthesis of free-standing, continuous and stable monolayer amorphous carbon^15,16^ has been reported. Similarly, the synthesis of very thin (3 nm) amorphous boron nitride (aBN) was recently reported^17^, and simulations have even suggested possible structures for atomically thin aBN^18–20^.

Revealing the structure of highly defective or even amorphous materials is not trivial. For graphene, Raman spectroscopy is the most commonly used method for this purpose^21–28^. However, it only allows probing the defect density at a spatial resolution of ca. 1 $\mu$m (which can be improved by two orders of magnitude using tip-enhanced Raman spectroscopy^24,26–28^). Additionally, it is poorly suited for distinguishing between different types of defects. At the same time, there are only a few studies where the atomic structure of point defects in hBN has been experimentally revealed^29–32^.

For obtaining atomic-resolution information, there are only two practical options: scanning probe microscopy and (scanning) transmission electron microscopy ((S)TEM). From these, (S)TEM can be more readily applied to samples on a large scale, however a notable downside is that the 2D material samples must be made freestanding. Furthermore, especially aberration-corrected annular dark field (ADF) STEM provides easily interpretable images of the physical and chemical atomic structure through the so-called *Z*-contrast.^33^. The main bottleneck for statistically relevant structural analysis of disordered 2D structures with STEM-ADF imaging is the small field-of-view (FOV) required for atomic-resolution images, typically containing only a few nm per spatial direction. However, this can be partially overcome with automated image analysis techniques. Indeed, automated image analysis has become an active research field of its own over the past years. The applied approaches range from a simple thresholding approach^14^ to more sophisticated methods enabled by increasingly efficient machine-learning algorithms. These include various neural network architectures that have already been used to analyze (S)TEM images (see for example Refs.^34–39^), autonomously yielding segmentation of atomic structure or enhancing images^35,37,38,40,41^ and classifying defects^34,36,39,42^. However, it is important to also ensure a bias-free collection of a sufficient number of atomic-resolution images for the automated analysis, as we showed recently for defect-engineered graphene^43^.

In this study, we apply the same methodology to graphene and hBN samples with different amounts of disorder, starting with well-separated point defects and ending with significantly overlapping defects. Each sample is imaged semi-automatically, and the images are analyzed with convolutional neural network-based methodology to reveal the atomic structure. We further discuss the applicability of the used approach and possibilities for its improvement in the future. Overall, our results show that automated imaging and image analysis together allow unambiguous characterization of even highly defective 2D materials, which reveals a marked difference in the response of the two materials to increasing disorder: graphene accommodates the increasing defect density by incorporating non-hexagonal rings into its structure, whereas in hBN the disorder manifests simply as empty lattice sites. This indicates that creating a truly amorphous atomically thin BN structure is a formidable challenge, in stark contrast to atomically-thin 2D amorphous carbon membranes.

## Methods

### Sample preparation

Graphene and hBN samples were fabricated using chemical vapour deposition (CVD)-grown commercially available materials by Graphenea© (graphene) and Graphene Supermarket (hBN). Graphene samples were provided on a sacrificial polymethyl methacrylate (PMMA) layer (Easy Transfer graphene) and were transferred onto a SiN TEM grid via a liquid transfer method using deionized water as the carrier liquid. Following the transfer onto the substrate the graphene was heated on a hot plate at $150^{\circ }$C for 1 h. The PMMA sacrificial layer was then removed via an acetone bath at $50^{\circ }\textrm{C}$ for 1 h, after which the grid rested in isopropyl alcohol (IPA) at room temperature for another hour. hBN was provided on the Cu growth substrate and was transferred onto a Au Quantifoil TEM grid (with a periodicity of 2 $\mathrm {\mu m}$ and a hole size of 2 $\mathrm {\mu m}$) following the procedure described in Refs.^30,31^: Cu was etched in a bath of 1.5 g of iron chloride mixed with 200–240 g deionized water for 47–72 h, after which the sample was washed in three cycles of water and IPA, for ca. 1 min each. For one hBN sample, the Quantifoil was placed onto a Si TEM chip with a perforated SiN window (hole size of 1 $\mu$m) where it was adhered by putting a small drop of IPA on it and heating on a hot plate at $150^\circ$C for 15 min. After that it was mechanically delaminated from the Au grid. For this sample the suspended area for the experiments can be found where the holes of the Quantifoil and the SiN overlap. The other hBN samples were transferred onto Quantifoil as described above.

### Sample cleaning

Samples were loaded into an interconnected ultra-high vacuum (UHV) system^44^ through a loadlock where they are baked in vacuum at ca. $150^{\circ }$C for over 10 h. Within the vacuum system, the samples can be transported between the microscope and the sample alteration chamber where they can be exposed to low-energy ion irradiation. To guarantee that a clean sample is irradiated, different methods were used for sample cleaning depending on the availability at the time of each experiment. Graphene samples were cleaned using a 6 W continuous wave diode laser with a wavelength of 445 nm (LASERTACK) with a spot size of 0.3$\times$1.5 $\hbox {mm}^2$ as described in Ref.^45^. The surface was illuminated at 17% of the maximum power for a duration of 10 min, where the power was set by changing the duty cycle. With these parameters, not all of the surface contamination is removed, but large enough atomically clean patches are created for acquiring statistically meaningful data. One hBN sample was cleaned by applying a laser pulse in the column of the scanning transmission electron microscope used for imaging, similar as in Ref.^32,46^; the exact parameters are described in Ref.^32^. The other (highly-defective) hBN sample was annealed in a UHV heating stage at $500^{\circ }$C for ca. 1 h. This was sufficient to remove static contamination and get reasonably clean areas (up to a hundred nm in diameter).

### Ion irradiation

The samples were exposed to low-energy ion irradiation using an SPECS ECR-HO microwave plasma source operated in hybrid mode similar to Refs.^32,43^. All irradiations in this work were conducted at room temperature using Ar ions. Comparing the different irradiation parameters directly is impossible as the plasma source needed to be maintained and modified during the course of the experiments. The pressure in the chamber varied between $3\times 10^{-6}$ and $7.3\times 10^{-6}$ mbar and the irradiation times varied between 2 and 30 min. In all cases, the plasma was stabilized for at least 10 min, after which the sample was inserted into the ion beam path. At the position of the sample, the ion plasma cone diameter is estimated to be several cm. Considering the TEM grid diameter of 3 mm, we assume an approximately constant flux throughout the entire sample. We expect the energy of the ions to be below 1 keV for all irradiations.

### Microscopy

After irradiation, the samples were transported to the Nion UltraSTEM 100 microscope through the UHV tubes of the vacuum system. There, they were imaged under UHV ($10^{\mathrm {-10}}$ mbar) using the medium angle annular dark field (MAADF) detector with a semi-angular range of 60–200 mrad. Graphene was imaged at 40 kV whereas hBN was imaged at 60 kV. The convergence semi-angle was 30 mrad, and the beam current at 60 kV in this mode is typically about 20 pA. Automated image acquisition^47^ was used to acquire atomic-resolution images over large areas on the order of hundreds of nm per lateral dimension, similar to Ref.^43^. The images were nominally 5 nm $\times$ 5 nm for graphene and 4 nm $\times$ 4 nm for hBN. For these acquisitions, each image contains 512 $\times$ 512 pixels, and on each pixel the scattered intensity is accumulated typically over 16 $\mu$s. For the semi-automatic imaging algorithm, four corner points of the area of interest are selected, at each point the sample is brought to focus by a combination of adjustments in the stage height and minute changes in the high tension (fine focus). The parameters of the stage height and focus are saved together with the (*x*, *y*) positions of the respective corner. With this information, the algorithm executes a bilinear interpolation in order to compensate for lateral sample slope, such that the sample is in focus in every element of the imaged array. The stage moves along a serpentine path between the points and acquires images at every stage position. In order to avoid overlaps in the imaged areas as a result of imprecision in the stage movement, we used an offset with a size of 1–3 image frames in between each two image acquisitions. To circumvent imaging artefacts due to residual stage movement after each translation, a sleep time of 2 s was chosen between the end of translation and the start of the actual ADF scan. The datasets containing hundreds of atomic-resolution images were saved on disk for further analysis. During the semi-automatic image acquisition, minor adjustments were typically made manually to correct for aberrations (focus, astigmatism, coma).

### Autonomous image analysis

An automated image analysis approach was used to process the hundreds of images in each dataset. The approach consists of three steps: neural network preprocessing, core neural network processing, and postprocessing of the core output. Images are preprocessed by masking out contamination and replacing the contaminated areas with the mean value of the non-contaminated areas. This is performed by a neural network, which segments the image into contaminated and non-contaminated areas.

170 randomly selected experimental graphene and hBN images images were used for the training of this preprocessing model. The defect regimes in the training images ranged from pristine to medium-defective. A small subset of these images were first segmented manually by selecting a threshold. This subset was used to training a linear neural network meant to serve as the preprocessing unit, however it was discarded due to its insufficient performance. The idea behind the linear neural network was to map an input of histogram values of the image to an output of a single value threshold which is then used to segment the image. This linear neural network was however used to accelerate the creation of the full training set for the final preprocessing model, which is a convolutional neural network. If the linear neural network segmented a selected image correctly, its output was included in the full training set. Where it did not yield satisfactory results, manual segmentation was employed as described before. The underlying architecture of the final preprocessing model is the U-Net architecture^48^, and all machine learning used here relies on the Python package PyTorch^49^. The masked but otherwise raw images are then given to the core processing unit, which is a fully convolutional neural network, with an underlying U-Net architecture and the inclusion of rotational invariance as provided by the package e2cnn^50^. It consists of two models, trained on simulated STEM-ADF images of the two materials.

The simulated images were generated using *ab*TEM^51^ based on underlying atomic models created using the Atomic Simulation Environment (ASE) Python package^52^. First, pristine atomic models were generated, which were assigned random small perturbations to the atomic positions, after which carbon contamination and a random number of vacancies were added.

For graphene, the underlying atomic structure was modelled with molecular dynamics to represent the bond rearranging, similar to Ref.^43^. Due to the fact that, based on our experimental images, hBN does not exhibit bond rearranging, molecular dynamics were not employed in the generation of the hBN training set. Ca. 5% of the hBN training dataset included pores, based on their occurrence in prior (not highly-defective) experimental observations. Therefore, the training data for both models included mostly only the low- and medium-defective regime.

These atomic models were used to simulate STEM images for the training set. Random scan distortions and diffuse contamination were applied to the images, and noise was added in the training process. Ca. 500 training images were used for each model. The neural network output for each input image consists of an atomic density segmentation map, which is used to determine the atomic positions. The atomic positions are then further analysed in the postprocessing step.

In the postprocessing step for graphene, the atomic positions were used to generate a stable Delaunay graph^54^, from which defects are extracted by analysing the deviation of the graph from an ideal graphene lattice graph. The defect area is calculated from the enclosed areas of the graph. For hBN, the core processing does not classify defects, resulting in the identification of atomic positions corresponding to an intact hBN lattice even in regions where the input image shows none. A stable Delaunay graph is calculated in this case as well. The atomic positions need to be evaluated by a specific criteria to be able to autonomously differentiate between atomic positions correctly assigned to the lattice and those incorrectly assigned to non-lattice regions. For this purpose, defect classifiers are employed to determine whether an atomic positions is a lattice point or defect. Atomic species assignment is performed by calculating the mean intensities of each sublattice, calculated from the graph. The defect area is then calculated by multiplying the number of connected defects with the single atom area in hBN. For both materials, atomic positions falling under contamination are discarded.

## Results and discussion

**Fig. 1:**
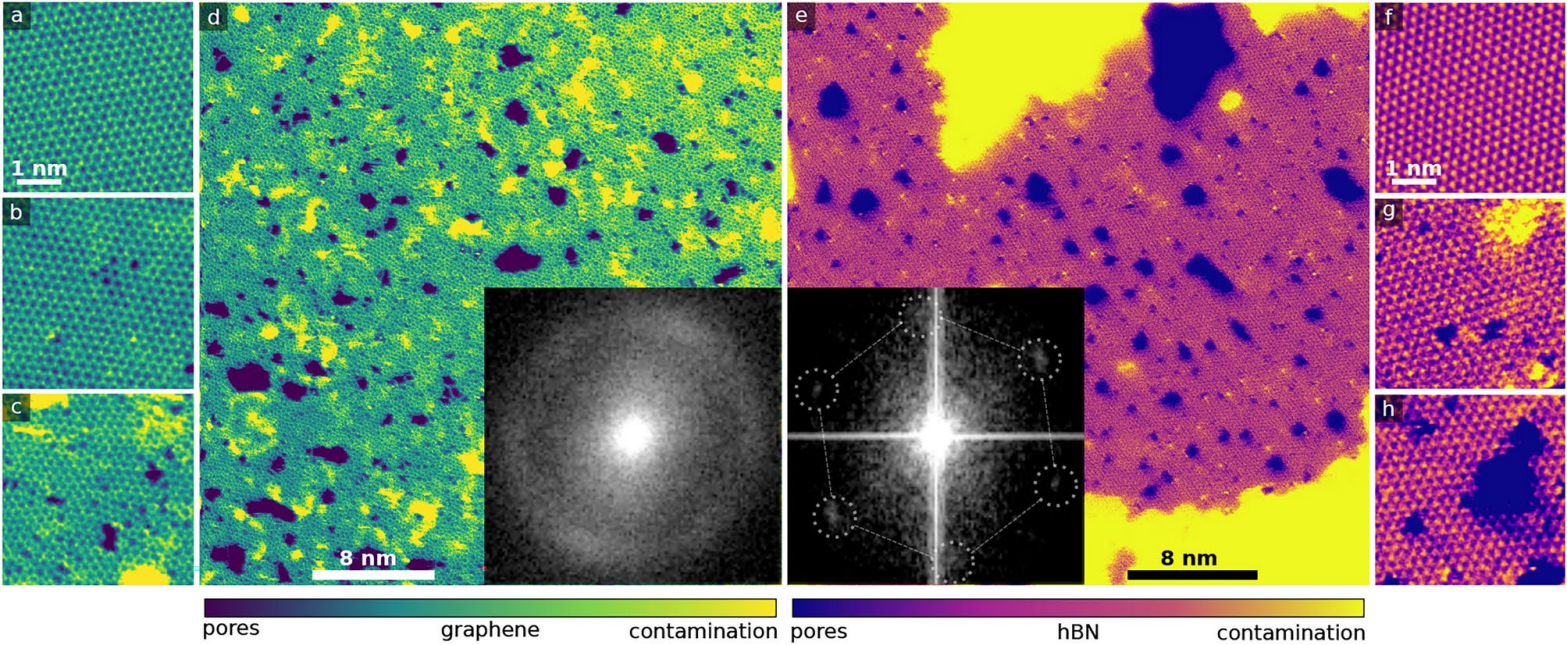
STEM-ADF images of graphene and hBN samples with an increasing defect density. (**a**) Pristine graphene, (**b**) Medium-defective graphene, (**c**) Highly-defective graphene. (**d**) Overview image of highly-defective graphene with many pores. The inset shows a fast Fourier transform (FFT) of the image. (**e**) Overview of highly-defective hBN, with an inset showing the corresponding FFT. The overlay indicates the positions of the two FFT spots, including two which can not be easily distinguished due to overlap with the vertical stripes. (**f**) Pristine hBN lattice, (**g**) Medium-defective hBN. (**h**) Highly-defective hBN. All smaller images have the same scale. The colormap applied on the graphene images is ’viridis’ and that on the hBN images ’plasma’.

Free-standing graphene and hBN samples were prepared from commercially obtained material as described in the Methods section. They were either imaged and analyzed directly as control samples or after being irradiated with low-energy Ar ions. After cleaning and irradiation, the samples were imaged semi-automatically with a procedure similar to that in Ref.^43^. The graphene samples were imaged at 40 kV, the hBN samples were imaged at 60 kV. In the case of all samples except for highly-defective hBN, we thus may assume that the role of the electron beam was negligible based on the established displacement cross sections under low-energy electron irradiation for hBN^31^ and graphene^53^. For that one particular sample, we noticed that the electron irradiation during imaging led to an increase in the size of the pores that had been created by the Ar ion irradiation. This is however not of critical concern here, since we are mainly interested in determining the resulting atomic structures rather than in analyzing the relationship between the irradiation parameters and the structures in detail. Samples with different defect densities were created by varying the irradiation time. The total number of atomic resolution images per dataset varied from 350 to 1000, with a nominal field of view of either 4 or 5 nm. Example images are shown in Fig. 1.

There is a marked difference between the graphene and hBN structures, immediately obvious already from these example images: at the highest defect density, graphene contains disordered structures of many non-hexagonal carbon rings (amorphization), which lead to the diffuse ring in the fast Fourier transform (FFT) shown as an inset in Fig. 1d. In contrast, defective hBN keeps its original crystal structure even at the highest defect density, as can be seen from the distinct spots in the FFT overlay in Fig. 1e. There are also practically no non-hexagonal rings.

**Fig. 2:**
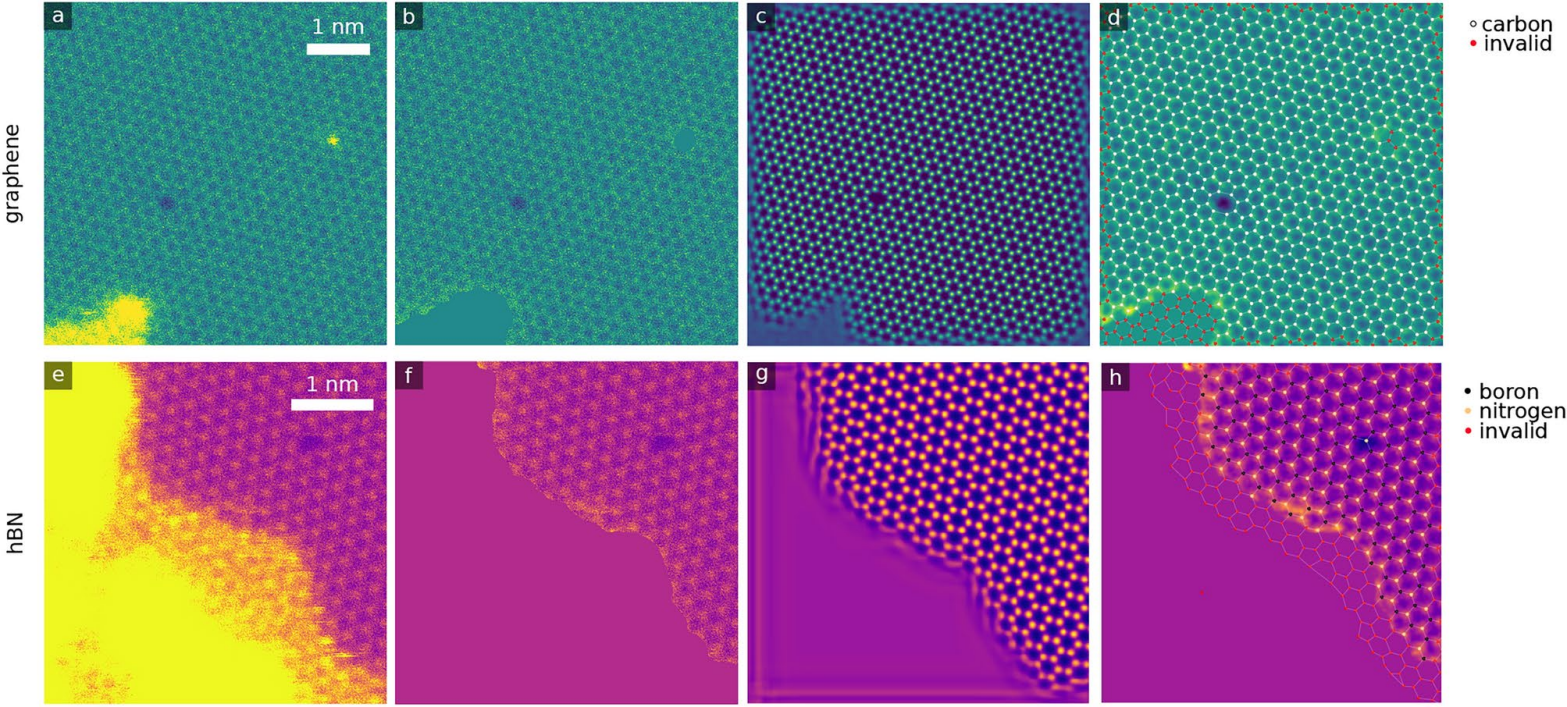
Autonomous image processing. (**a**–**d**) Autonomous image processing using the graphene model and (**e**–**h**) hBN model. (**a**,**e**) Raw input STEM-ADF image. (**b**,**f**) Neural network preprocessing, masking the contamination. (**c**,**g**) Outputs of the core neural network processing unit, producing atomic density images. Note that the graphene model in panel c accounts for defects, while the hBN one in panel g outputs atomic positions also in defect regions. (**d**,**h**) Post-processing of the output, calculating discrete atomic positions from the atomic density images, and classifying them as valid or invalid (contaminated, boundary). For panel h, the boron and nitrogen sublattices are identified based on local intensities at the atomic positions.

To characterize the atomic structure and defects in detail, we employ a multistep autonomous neural network approach. The approach is based on autonomous detection of atomic positions in the images, which forms the core processing unit of the approach, as described previously in Ref.^43^. The process is schematically illustrated in Fig. 2. In brief, an input image undergoes three steps in the autonomous analysis pipeline: preprocessing, core processing and postprocessing. Hydrocarbon contamination, which appears as bright diffuse contrast in the STEM-ADF images (see Fig. 2a,e) would skew the results and must be masked before the actual analysis. This is performed by a neural network preprocessing step, which yields an output that can be seen in Fig. 2b,f. The neural network was trained on experimental images to return binary masks segmenting the image into contaminated and non-contaminated areas. These masks were used to replace the contaminated area with a mean value of the non-contaminated area.

The preprocessed image is passed onto core processing, which generates an atomic density image as seen in Fig. 2c,g. For this step, we use separate models for graphene and hBN, which perform differently: for graphene, the model was trained on simulated images based on molecular dynamics simulations to account for rearrangements within the lattice as seen above. For hBN, the model was trained on simulated images based on the perfect lattice, as bond rotations are not expected in this case based on the experimental images. As a result, the hBN model also outputs atomic densities where there are no atoms in the image, as can be seen in Fig. 2h. Due to the different behavior of the two models, the postprocessing also differs, and will be described separately.

The postprocessing in graphene is based on the analysis of the stable Delaunay graph^54^ calculated from the atomic positions. Each polygon in the graph is first evaluated on its ring size, where non-hexagons are classified as defects. The defect is further evaluated for validity, where it is valid if it is within the image boundaries and if it is closed. The postprocessing yields the enclosing area of valid defects, the missing atoms, as well as the ring sizes. This approach performs well for low (pristine) and medium defect densities in graphene and requires no manual intervention. In the high defect density regime, due to the nearly full amorphization of the lattice, most defects will not be closed within the imaged field of view. In this regime, it is useful to evaluate the lattice polygon ring sizes as an indicator of defectiveness. However, while the model still outputs accurate atomic positions in images where the lattice is highly disordered to amorphous, it fails to account for larger pores (ca. 10 or more atoms missing), rarely but occasionally yielding atomic positions within them, which adds an uncertainty otherwise not present in the medium defect density regime analysis. The preprocessing neural network also exhibited issues with images containing large pores, yielding inaccurate contamination segmentation maps. This is because the preprocessing model assumes the darkest intensity to be non-contaminated, which could be traced back to its training set and training process: in order to reduce computational time and complexity of the input images, the training set and all inputs are scaled down to 64$\times$64 pixel images. The model, therefore, failed to recognize the lattice features and instead focused solely on evaluating the intensities. Since the training set did not include porous images, when analyzing the highly defective regime in graphene, contamination was masked out using a single threshold, manually chosen for each individual microscopy dataset (collection of images recorded within the same automated acquisition). This yields results similar to the neural network contamination segmentation.

For hBN, the postprocessing involves multiple steps: first, invalid atomic positions are removed (positions at the image boundary or within contamination). As the model places atomic positions within defects, which is useful to easily extract atomic species, it also necessitates a defect classifier, analyzing each valid atomic position. To test the analysis method, datasets with varying data quality were recorded. This, however, necessitated the creation of four different defect classifiers: one based on Gaussian blurring and local intensities, one based on a manually trained machine-learning approach requiring separate training for each dataset, and two different ones based on a comparison between local atomic intensities and local void intensities, calculated from positions in the middle of the lattice hexagon. A stable Delaunay graph is, in this case, used only to find the sublattice for the two atomic species, which are assigned based on the local intensities. As hBN does not exhibit bond rotations, the graph and polygon shape analysis was not employed. The graph is also used to identify connected defects to determine their size. Lattice positions which are not clear enough are marked as invalid. This is assessed by calculating the root mean square deviation from the ideal lattice of each point (in percentage), where positions with over 3% deviation from the ideal lattice are discarded. Such atomic positions generally occur in images where strong imaging aberrations are present, or when atomic positions were placed in contaminated (masked) regions. Therefore, defects with unclear neighbors (<3) are considered invalid, which occurs when a defect is covered by contamination or is in a highly aberrated region.

The postprocessing for hBN then yields the number of connected missing atoms and their species. While the core model output itself performed similarly to the graphene model, yielding accurate atomic positions across varying defect concentrations (excluding the different behavior regarding defects), it does not accurately evaluate pores. It does not place any atomic positions in porous regions as it does for smaller defects, instead yielding highly disordered points at the pore edges. Therefore, for the highly-defective hBN, we make an estimation of the local lattice damage based on the imaged area and the porous area. The preprocessing neural network also fails for porous hBN, therefore the contamination masking was the same as described for highly defective graphene, and the porous areas are calculated using a manually selected threshold in the same manner. One threshold for porous intensities and contamination intensities is used for a single microscopy dataset, as the variation of these intensities in individual images is negligible. The porous areas do not include atomic vacancies below a missing number of 2–3 atoms, due to their higher intensities, which cannot be accounted for in a single threshold value. The ratio of porous area to valid area (which includes the porous areas) in the highly defective hBN then yields a value of 0.156±0.093 (assuming a confidence interval of 95%).

**Fig. 3:**
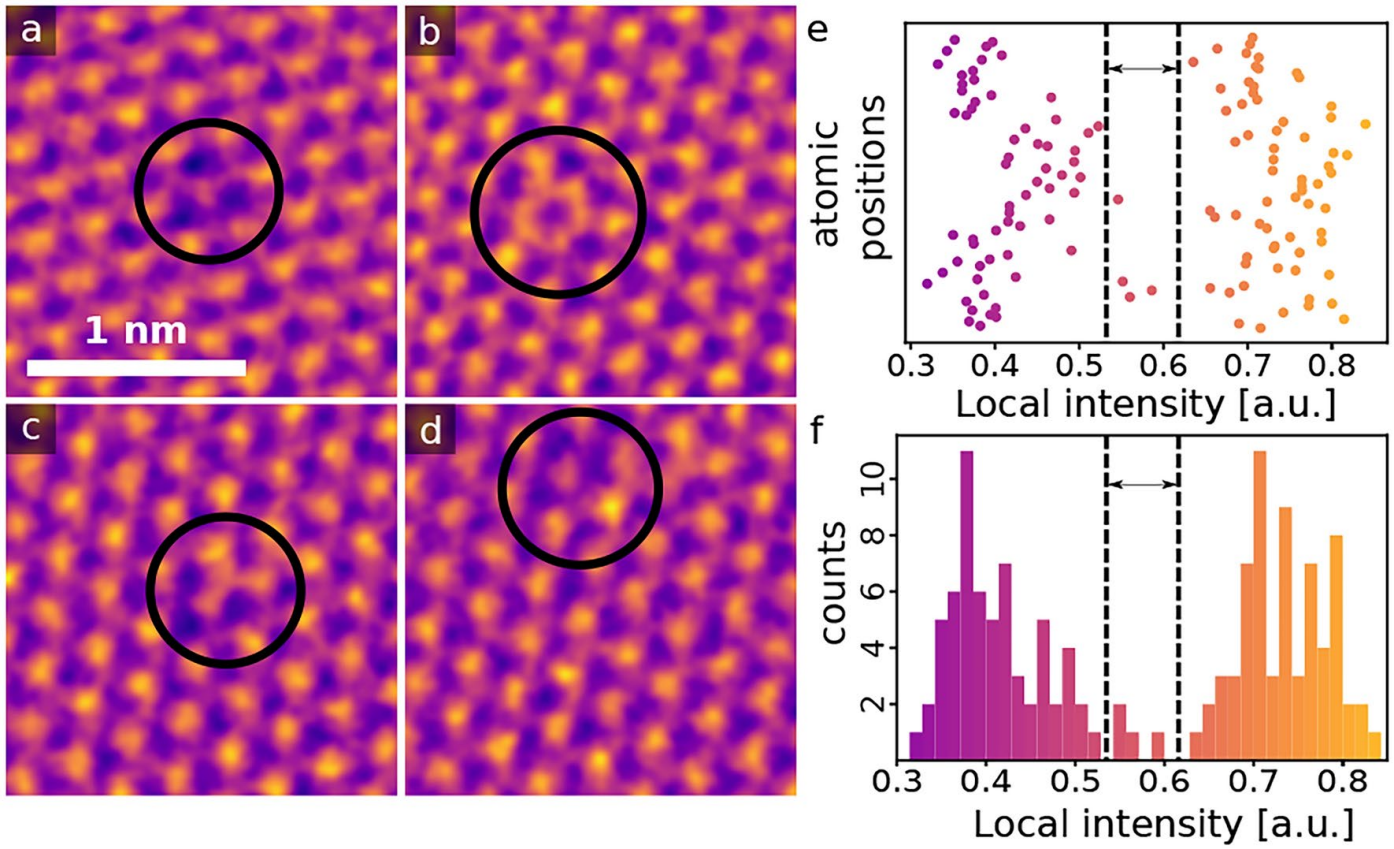
Filled defects in hBN. (**a**–**d**) STEM-ADF images of possible heteroatom substitutions in hBN. (**a**) A single carbon impurity, occupying a nitrogen site. (**b**) Hexagonal carbon ring. (**c**) Multiple carbon impurities. (**d**) Heavier impurity close to a carbon impurity. (**e**) Intensities of atoms within and around the black circles in panels **a**–**d**. (**f**) The corresponding histogram. Intensities between the two peaks likely correspond to carbon atoms.

We note that the defect analysis as described here only corresponds to non-hexagonal rings and empty lattice sites. However, the datasets also contain images where impurity atoms are embedded in an otherwise non-defective lattice. Examples of impurity atoms in hBN are shown in Fig. 3a–d. While these examples are from a dataset collected from a non-irradiated hBN sample, similar impurity atom sites are also present in the dataset for defective hBN and graphene samples. For clarity, we show in Fig. 3e,f intensities for each of the atomic sites and the corresponding histogram, which highlights the possible carbon impurity intensities as a small peak between the boron and nitrogen intensities. While we have not studied filled defects systematically, in most observed cases the impurity atoms seem to preferentially fill nitrogen sites in hBN.

**Fig. 4:**
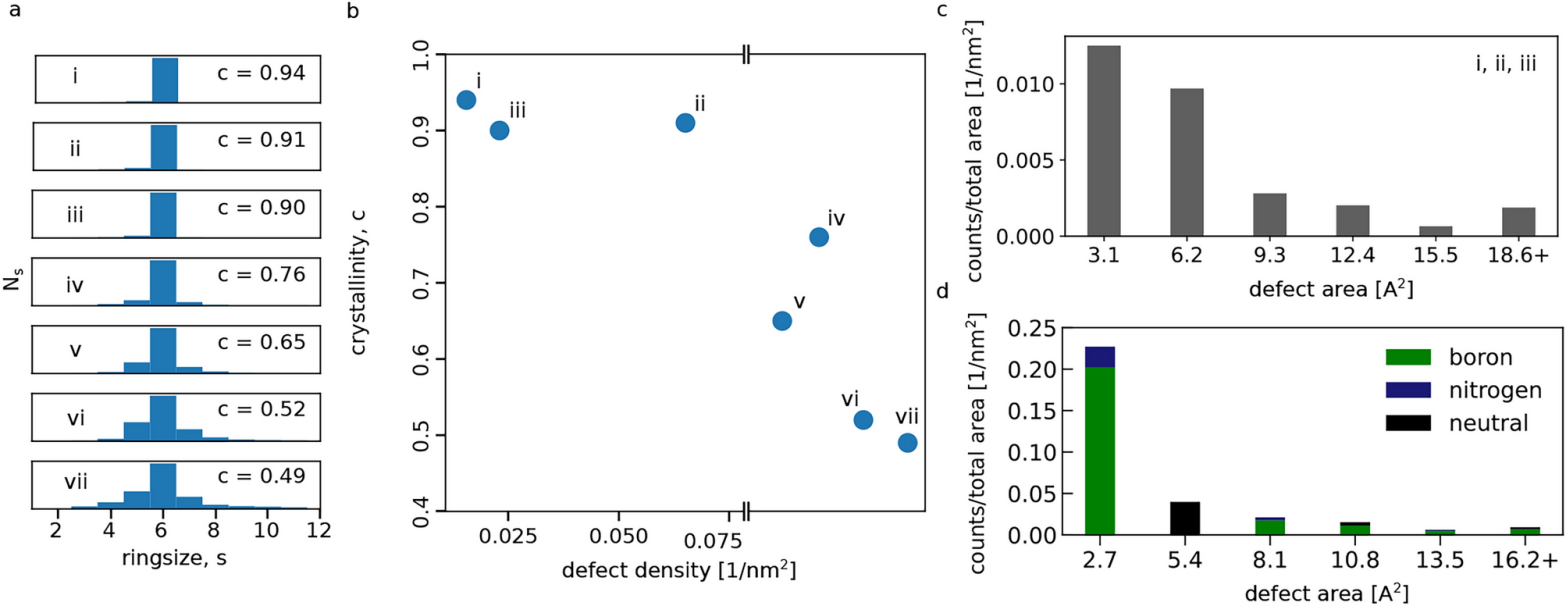
Defect shapes and sizes. (**a**) Ringsize *s* distributions for medium- to highly-defective graphene (top to bottom). Number of non-hexagonal polygons increases for higher defect densities. Each dataset is labeled by a Roman numeral to ease the comparison between the different panels. (**b**) Relation between crystallinity *c* and defect density in graphene. Datapoints within the graph stem from the medium-defective regime, where most detected defects were closed within the images. Datapoints plotted outside the *x*-axis range stem from the highly-defective regime, where the defect density could not be meaningfully calculated due to large disorder in the lattice, yielding little to no closed defects. These datapoints are ordered from the largest (left, v) to the smallest number of closed defects (right, vii) per valid area. (**c**) Defect areas of medium-defective graphene calculated from the number of missing atoms plotted against the number of defects per total analyzed area. The last bar contains all defects with the given size or larger. (**d**) Defect areas of medium-defective hBN calculated from the number of missing atoms against the number of defects per total analyzed area. The color indicates which species is the majority of the missing atoms, where neutral denotes equal numbers of missing boron and nitrogen atoms. The smallest defects in both cases correspond to single vacancies, and the second smallest to double vacancies, etc.

In Fig. 4a, ringsize *s* statistics are presented for medium- to highly-defective graphene. A high defect density leads to the formation of a considerable number of non-hexagonal carbon rings in the lattice, which is not observed for hBN. In Fig. 4b, the crystallinity *c* of these samples is plotted against the defect density. The crystallinity is calculated as the number of hexagons divided by the number of all polygons, similar to Ref.^14^. Due to the large defect sizes, as described previously, the defect density could not be calculated for the highly defective regime, which are the four datapoints plotted outside the *x*-axis from left to right in order of decreasing number of closed defects. In Fig. 4c,d the calculated defect sizes based on the autonomous image analysis are shown for medium-defective graphene and hBN, respectively. The defect areas are calculated by multiplying the number missing atoms by the minimum area occupied by one atom. The counts (number of defects) were normalized using the total analyzed area of the dataset for better comparison between the two materials. For hBN the total analyzed area is calculated by multiplying the number of detected valid atomic positions by the minimum area and, in the case of graphene, using the FFT-calibrated field-of-view, disregarding contaminated areas. The results for hBN show a clear preference for B vacancies, which may be due to an unequal filling of defects by impurity atoms, which is not taken into account here.

Altogether, the two models were both successfully applied for the low- to medium-defective datasets. Therefore, while this approach has the benefit of being based on synthetic data, therefore relying less on human biases induced in shaping and creating labelled training sets, it remains limited to the features included in its training set with little or no capacity for extrapolation. While the training set can be expanded to cover all types of data, as encountered here, this requires that all features that appear in the experimental data are known *a priori*, so that they can be included. Additionally, significant amount of human time and computational resources must be spent to develop and include new features to the training set whenever they are encountered in the experimental data. This might be avoided with an alternative approach relying on enhancing input images, generating an ideal lattice fit to the input and evaluating the difference between the two, allowing to extract contamination, defect structures and all other non-lattice features without synthetic training data based on atomistic simulations. However, we have not encountered such an approach in the literature for the scale of features relevant for our data. It is also worth noting that the U-Net architecture, as used here, was developed to segment medical images, which might be of redundant complexity for the analysis of lattice images, leading to longer training times and possible overfitting.

## Conclusions

Different defect regimes of freestanding monolayer graphene and hBN were studied by introducing defects at varying concentration using low-energy ion irradiation, from the lowest defect regime of pristine material to the highly-defective regime and complete amorphization. Thousands of STEM-ADF images were semi-automatically acquired for accurate representation of the defect statistics. These result reveal a marked difference in how the two materials respond to increasing disorder. In graphene, bond rotations facilitate the amorphization of the lattice by introducing a larger number of non-hexagonal rings, whereas in hBN, disorder manifests only as empty lattice sites. Based on these results, it is unclear whether a truly amorphous atomically thin BN can exist. We further showed that automated atomic-resolution image acquisition combined with neural network-based automated image analysis is a powerful technique that allows for the characterization of 2D materials at all levels of disorder. However, a fully automated method that would be applicable to any experimental dataset does not yet exist, as shown by the required manual training and lack of extrapolation capacity of the models used here. Nevertheless, already at this stage, the method is a crucial addition to the analysis toolkit, which reduces human bias both from selecting the imaged areas as well as identifying atomic structures from experimental images. At the same time it also reduces the time required for image analysis.

## Data Availability

The datasets generated and/or analysed during the current study are available in the University of Vienna Phaidra repository at Ref.^55^.
